# 

**Published:** 1891-02-07

**Authors:** 


PRICE ONE PENNY.

No. 228, Vol. IX.—February 7th, 1891.

[Registered at the General Post Office as a Newspaper for transmission in the United Kingdom and Abroad.]

WITH EXTRA NURSING SUPPLEMENT.
Per Annum, 6s. 6d., post free.
Monthly Parts, 6d.; post free 8d.
Ditto per Annum, 8s., post free.

### THE HOSPITAL

GLASGOW WESTERN INFIRMARY — ST THOMAS'S HOSPITAL — NORFOLK AND NORWICH HOSPITAL

A WEEKLY JOURNAL OF

### Science, Medicine, Nursing and Philanthropy.

SATURDAY, FEBRUARY 7th, 1891.

**Medical Women at the Johns Hopkins University** - 283
**Passing Topics.**—A Critic Criticised - 284
**The Doctor's Armchair.**—A Dream: Perhaps a Delusion 285
**The Microscope in Medicine.** By Frank J. Wethered, M.D.—IV. "Cutting Sections," 286; **Everybody's Page** 287
**Notes and News** - 288
**Restrictions on Gifts to Charities** - 289
**Scraps and Gleanings** - 289
**Annotations.**—The Nurses' Co-operation—One-Roomed Life in London—Anti-Vaccinators at Tunbridge Wells - 290
**Working Women in Large Towns.**—XIX. What the General Public May Do - 294
**The Practitioner's Mirror and Retrospect:**—
The Post-Graduate Course at the Brompton Consumption Hospital.—On the Treatment of Cough - 291
Medicine.—Cardiovascular Vertigo—Primary Acute Encephalitis—A New Cause of Tabes—Reflex Epilepsy from Dental Caries—Diabetic Coma, 292; Anomalous Ataxies 293
Therapeutics.—Euphorine - 293
**The Practitioners' Bookshelf.**—Nasal Obstruction and Mouth Breathing—Electro-Therapeutics—Medical Bookkeeping - 293
**The Student of Medicine.**—Appointments—Vacancies—Presentations—Pass Lists—Events for the Month.

EXTRA SUPPLEMENT—THE NURSING MIRROR.

**En Passant.**—Frome Nurses' Home—Pension Fund Presentation—Helping One Another—Short Items—The Beating of Patients—Coventry Nursing Association—Another Fever Hospital Scandal—Hospital Scandals - ci

**Lectures on Surgical Ward Work and Nursing.** By Alex. Miles, M.B. Edin., C.M., F.R.C.S.E.. Lecture XII.—Management of Surgical Operations - cii

**Nursing Medals and Certificates**—Liverpool - ciii

**Presentations** - ciii

**For Reading to the Sick.**—To the Young - ciii

**Nurses on Their Travels.**—Lady Roberts' Nurses in India civ

**The Lords' Committee—Appointments** - civ

**Everybody's Opinion.**—In Darkest England—Consumption Cures - cv

**National Pension Funds for Nurses.**—A Help and Consolation.—**Notes and Queries** - cv

**Off Duty.**—Nurse Hilary.—**Where to Go** - cvi

**Amusements and Relaxation** - cvi

